# Climate Change Threatens Coexistence within Communities of Mediterranean Forested Wetlands

**DOI:** 10.1371/journal.pone.0044727

**Published:** 2012-10-08

**Authors:** Arianna Di Paola, Riccardo Valentini, Francesco Paparella

**Affiliations:** 1 Department for Innovation in Biological, Agro-Food and Forest Systems, University of Tuscia, Viterbo, Italy; 2 Division of Impacts on Agriculture, Forest, and Natural Ecosystems, Euromediterranean Center for Climate Change, Viterbo, Italy; 3 Department of Mathematics and Physics Ennio De Giorgi, University of Salento, Lecce, Italy; Ohio State University, United States of America

## Abstract

The Mediterranean region is one of the hot spots of climate change. This study aims at understanding what are the conditions sustaining tree diversity in Mediterranean wet forests under future scenarios of altered hydrological regimes. The core of the work is a quantitative, dynamic model describing the coexistence of different Mediterranean tree species, typical of arid or semi-arid wetlands. Two kind of species, i.e. Hygrophilous (drought sensitive, flood resistant) and Non-hygrophilous (drought resistant, flood sensitive), are broadly defined according to the distinct adaptive strategies of trees against water stress of summer drought and winter flooding. We argue that at intermediate levels of water supply the dual role of water (resource and stress) results in the coexistence of the two kind of species. A bifurcation analysis allows us to assess the effects of climate change on the coexistence of the two species in order to highlight the impacts of predicted climate scenarios on tree diversity. Specifically, the model has been applied to Mediterranean coastal swamp forests of Central Italy located at Castelporziano Estate and Circeo National Park. Our results show that there are distinct rainfall thresholds beyond which stable coexistence becomes impossible. Regional climatic projections show that the lower rainfall threshold may be approached or crossed during the XXI century, calling for an urgent adaptation and mitigation response to prevent biodiversity losses.

## Introduction

The Mediterranean climate is determined by complex interactions between global and regional circulation patterns involving both the oceans and the atmosphere. The increased greenhouse gas emissions make the Mediterranean a region vulnerable to climatic changes [1], [2]. Indeed, this region has experienced large climate shifts in the past [3] and it has been identified as one of the most prominent hot-spots in future climate change projections [1], [4]. The particular features of such a climate allow for the establishment of a unique Mediterranean biome, characterized by plant species usually adapted to dry and hot summer climate [5]. Nevertheless, the Mediterranean basin has a large number of wetlands, for a total of over 4 million hectares [6].

The Mediterranean wetlands are of great importance because they are hot spots of biodiversity, represent the basic land refuges for bird migration, and they contribute to maintain a complex equilibrium with coastal and estuarine ecosystems [7]. The impact of climate change on the biodiversity and the coexistence of species may be even more important in those wet-adapted ecosystems than on drylands, due to the predicted changes on the hydrological cycle for the Mediterranean region.

The vegetation in Mediterranean climates is typically sclerophyllous and ever-green, adapted to water stress during the dry summer period, and able to grow on infertile soils [8]. However, the availability of year-round moisture in swamps or near streams areas enables deciduous woody vegetation to occur in the riparian zone of Mediterranean-type streams in the Northern Hemisphere with equivalent species pairs occurring in different Mediterranean regions (e.g. Israel and California) [9]. Coexistence of different water-related species in Mediterranean woodlands and riverine floodplain, and in arid or semi-arid wetlands boundaries are documented in the literature [10], [11], [12]. Of particular interest are the coastal plain forests located behind the dunes and in the interdunal wet environments, that in the past had a wide distribution along the Italian coast. Currently they are fragmented and reduced in area, due to strong anthropogenic changes (sewage, drainage, cultivation, construction, tourism) [13], [11].

In coastal plain forests of the Tyrrhenian, the high availability of edaphic water allows the coexistence of sclerophyllous evergreen oak species (*Q. ilex* and *Q. suber*) with deciduos ones (dominated by a typical endemism of *Q. cerris* and *Q. frainetto* and with the significant presence of *Q. robur*) despite the meso-Mediterranean or thermo-Mediterranean climate location [11]. Moreover, small depressions (*pools*) are frequent in these forests, and play the role of water reservoirs for several weeks a year [11]. Despite their ecological value due to high biodiversity and endemism, very few scientific studies have addressed their ecology or the issue of their conservation [11], [13].

In this paper we develop a quantitative, dynamic model, that describes the coexistence between two groups of plants, that we name *hygrophilous* and *non-hygrophilous*, defined (in the next section) according to their distinct adaptive strategies for water stress. The ecological importance of distinct water management strategies for Mediterranean oak woodlands have recently been discussed [14]. However, to our knowledge, this is the first mathematical model that builds on those experimental findings in order to quantitatively explain the coexistence of species with differential adaptations to water stresses.

The model is embodied with a set of three ordinary differential equations and does not explicitly describe the spatial structure of the forest. We use the model to assess the resilience of coexistence to climate change. To this purpose we first identify the equilibria of the model and determine their stability (or, in other words, the range of parameters within which the biodiversity is preserved). Also we study the regime shifts that may occur when the hydrological parameters change according to the recent climate change scenarios [1], [2].

From a dynamical system point of view, regime shifts may be interpreted as mathematical bifurcations, triggered when an external cause changes one or more of the model parameters beyond a critical value. In some cases, the shift appears as a sudden, sharp, and dramatic change in the state of the system, as, for example, the shift from clear to turbid water in lake systems [15]. In other cases the transition in the state variables is more gradual, such as in the change from a grassy to a shrub dominated rangeland [16]. Dynamic models are probably the only option for describing and understanding complex ecosystem that are characterized by historical dependency, nonlinear dynamics, threshold effects, multiple basins of attraction, and limited predictability [17].

It is worth noting that how large number of competing plant species that coexist in ecosystems is a major unresolved question in community ecology [18]. Although spatially explicit models appear to be promising tools to understand some aspects of coexistence [19], [20], [21], [22] (see also [23] for the case of Mediterranean woodlands), an explicit modeling of space is not always necessary to explain it [24], [25]. For a review of the classical models and ideas proposed in order to maintain species diversity, see the work of Chesson [26]. However, whereas early coexistence theories focus on competitive exclusion of species with similar requirements [5], [27], [28], [29], recent ideas highlight that species diversity may be explained by a multitude of processes acting at different scales, and that similarities in competitive abilities may often facilitate diversity and coexistence [10], [30], [31], [32], [33]. The emerging evidence is that a general theory of coexistence does not exist, but distinct mechanisms (e.g. competition, facilitation, niche separation), highly depending on the characteristics of the system under study, may lead to distinct forms of species coexistence. Our model suggests a new mechanism of coexistence of tree species, related to the role of water. The model was devised to explain the observed coexistence in Mediterranean coastal forests. We believe that the basic idea may be adapted to other forests where distinct species cope with distinct strategies to water-induced stresses.

## Materials and Methods

We define the *water use efficiency* as the ratio between the plant growth rate and the plant water use, namely the water lost by transpiration. Drought and flooding are generally mitigated by several physiological acclimations that tune the water use efficiency or change the vulnerability to root asphyxia of plants [34], [35], [36], [37]. For the purpose of our discussion we consider two categories of plants: i) hygrophilous species, that tolerate the submersion of the roots but are drought sensitive, have low water use efficiency, and are not very able control transpiration in response to drought [35], [36]; ii) non-hygrophilous species, that are drought resistant but do not tolerate flooding, have higher water use efficiency, and are able to control water lost by transpiration in response to drought. Hygrophilous species have higher rates of 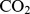 assimilation, which is generally reflected in higher maximal growth rates (attained at high soil water contents), compared to non-hygrophilous species [35], [38].

Here we argue that at intermediate levels of mean annual water supply, which do not benefit either of the two species, the dual role of water (resource and stress) results in the coexistence of these two kind of species.

### 0.1 Model formulation

Growth and functional response of plants to resource availability are generally regarded as the minimum set of factors that should be accounted for explain the observed coexistence of different plant types in forested wetlands [34]. An abstract dynamic model may therefore take the following form
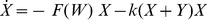
(1)

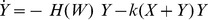
(2)

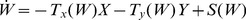
(3)where 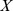 and 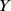 are the biomass densities of, respectively, the hygrophilous and non-hygrophilous species; 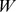 is the soil water content; the water-dependent functions 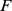 and 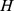 are net growth rates of the hygrophilous and non-hygrophilous species, expressed as biomass produced by one unit of biomass in one unit of time (we refer to them as ‘net’ because, as we detail below, we consider them as the sum of a metabolic growth rate and a water-induced mortality); the term 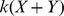 is the death rate (also expressed as biomass lost by a unit of biomass in a unit of time) due to competition for space (or light), which is proportional to the total biomass, with constant 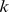; the water-dependent functions 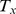 and 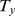 model the water loss to transpiration per unit of biomass; the function 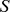 represents the algebraic sum of all biomass-independent sources and sinks of water. It is generally reasonable to assume 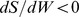 because some sinks (e.g. percolation) increase their flow rate for increasing soil water content, but sources (e.g. precipitation) are independent of it.

This abstract formulation should be thought of as valid within an intermediate range of water content. The lower end of the range must be above the permanent wilting point, and the upper end must be below the soil saturation concentration. The model values must also be taken as yearly averages, and are not representative of conditions that may occur for shorter times (such as seasonal floods or droughts). Outside the validity range of the model, even if the equations remain mathematically well-posed, other biological and ecological factors, neglected here, step into the picture. Therefore our model might not yield believable results in arid or permanently swampy environments.

The hygrophilous species' net growth rate 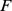 is assumed to be a growing function of soil water content 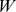: it attains its highest value at the upper end of the water content interval, and its lowest value at the lower end of the interval. The net growth rate 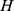 of the non-hygrophilous species is assumed to be a growing function of 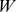 only up to some intermediate value of water content. For larger water contents it either decreases, or, at least, it doesn't increase as rapidly as 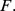


These assumptions are suggested by the dual role of water as a resource and as a stress factor. In order to separate the two effects, it is convenient to express the net growth rates as the algebraic sum of two terms, that we shall call “growth rates” (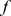, 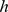) and “mortalities” (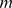, 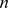).
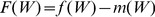
(4)

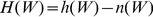
(5)The growth rates 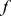 and 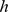 model the metabolic growth processes of plants. They are monotonically growing functions of the water content of the soil, and saturate at high water levels. An explicit expression for 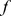 and 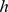 may be given by Michaelis-Menten functions (also known as Holling type II)
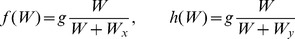
(6)where 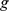 is the maximum growth rate, attainable in completely idealized conditions, which is taken to be the same for both species. This simplifying assumption is supported by the experiments of [39]. The coefficient 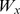 and 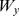 are half-saturation constants. In the introduction we have described non-hygrophilous species as drought-resistant due to their better water use efficiency. This suggests that they attain their maximum growth at lower water contents than the hygrophilous species. Therefore we may assume 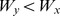, although we expect those coefficients to be numerically close to each other.

**Figure 1:**
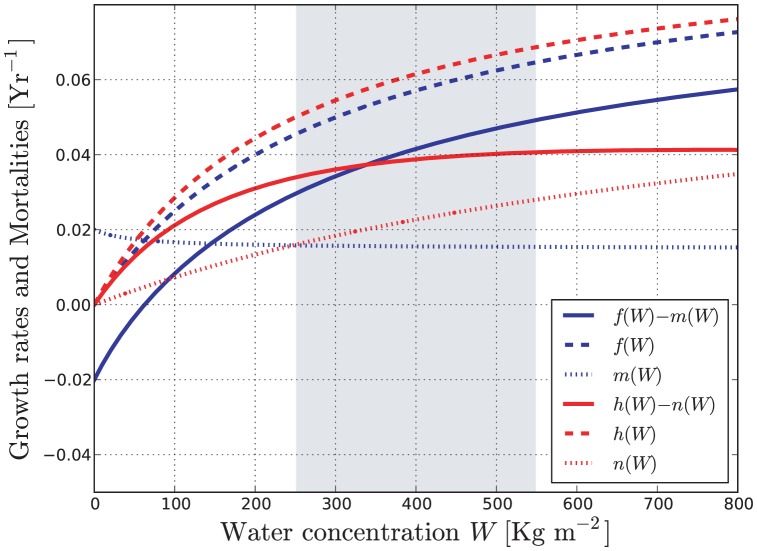
Water-dependent growth and mortality rates as in **equations (9**,**10**). Blue and red lines refer to the hygrophilous and non-hygrophilous species, respectively. The gray shaded area is the validity range of the model. Dashed lines are the growth rates, assumed to be proportional to transpiration; dotted lines are mortalities; solid lines are the resulting net growth rates 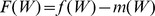 and 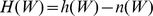. The parameter values used to draw the figure are those given in Table 1.

The water-dependent mortality functions 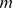 and 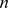 have distinct behavior for the two species. For the hygrophilous species, scarcity of water is a stress. Therefore we will take 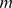 as a monotonically decreasing function of 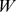. Viceversa for the non-hygrophilous species too much water is a stress factor. Therefore we take 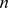 as a monotonically growing function of 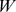. Below we shall use simple rational functions to express mathematically 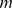 and 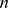 (see equations 9,10). We do not claim any particular physiological significance for this particular choice, which, we feel, is just a convenient way to give enough freedom to the possible shapes of the graphs of 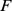 and 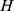, while limiting the number of free parameters. Figure 1 summarizes graphically the water-dependent growth and mortality functions of the model. Note that the curves representing the net water-dependent growth rates 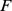 and 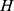 may or may not meet within the model validity region, depending on the physiological properties of the species being modeled.

Competition for space is modeled by a water-independent death term, proportional to the total biomass. Of course, the last term in equations (1) and (2) disregards completely a large number of complicated ecologic interactions which may be possible among plants. However, at least as a working hypothesis, it is best to assume that the mere crowding of the forest is the dominant factor affecting the death of trees. Note that, if the water content 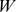 is externally kept fixed, and any one of the two plant species is absent (i.e. 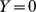 or 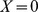) then the equation for the other reduces to a logistic equation with carrying capacity 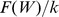 or 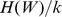. For simplicity, we keep the same coefficient 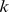 for both the hygrophilous and the non-hygrophilous species. This choice appears to be appropriate in the case study that we present in section 0.2. If necessary, distinct coefficients may be used, with minimal adjustments to the mathematical analysis presented below.

There is a strong correlation between the growth of plants and their transpiration [38]. The exact functional form linking the growth rate and the flux of transpired water is unknown, although there is evidence that it changes among different species, at different life stages of the plants, and it is affected by the local climate [40], [41]. However, experimental data for midlatitude forests show that a simple proportionality should be a reasonable approximation [40], [42]. Therefore, with suitable coefficients 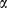, 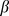, we may define
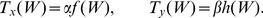
(7)


The transpiration rates in response to water variations can be thought of as a necessary cost associated with the metabolic growth. Because of the different strategies for water use efficiency, that cost is not the same for the two species: non-hygrophilous (or drought-resistant) species show lower transpiration rates than hygrophilous species both in drought and in wet conditions. In drought conditions, resistant species are able to control stomata better than hygrophilous species: this allows them to achieve relatively high photosynthetic rates with transpiration rates lower than their hygrophilous competitors [35], [36], [38]. In the presence of abundant water, hygrophilous species reach photosyntetic rates higher than those of resistant species, but at the expense of higher transpiration rates [34], [35], [38]. Therefore we may generally assume 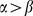.

The only source of water in the model is precipitation, , According to the observational evidence for midlatitude forests [35], [43], there also exists a non-negligible average evaporation rate 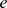 that is independent of plant transpiration. Finally we take the flux of water lost to deep percolation as proportional to the water content in the soil, with proportionality constant 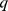. Therefore, the biomass-independent term 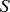 assumes the form
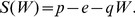
(8)


With the modeling choices discussed above, the equations (1,2,3) become
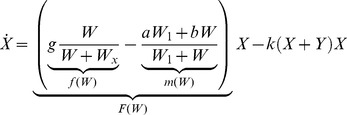
(9)

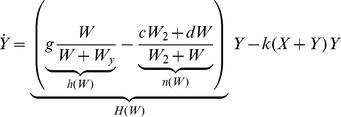
(10)

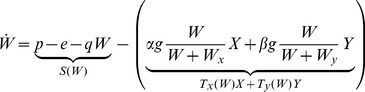
(11)where the biomass 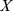 and 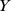, the water content 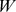, the half-saturation 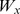 and 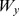, and the coefficients 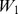 and 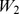 are given in 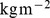; the optimal growth rate 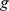 and the percolation coefficient 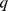 are in 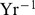; the coefficients 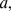 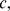 and 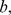 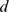 (formally the water-induced mortalities of the hygrophilous and non-hygrophilous species in the limit 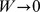, 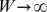) are also in 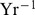; the coefficient of the biomass-dependent mortality 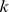 is in 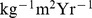; the precipitation 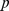 and evaporation 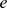 are in 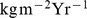; the proportionality constants 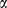 and 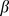 are dimensionless.

### 0.2 Choice of the parameters

In order to understand whether the model may be used as a quantitative tool for assessing the resilience of states of coexistence to climate change, we have sought a set of parameters that could approximately reproduce the available experimental data measured at two Mediterranean plain forests of Central Italy (Circeo National Park and Presidential Estate of Castelporziano).

Observational data about Mediterranean coastal plain oak forests can be summarized in the following points: (i) deciduous species (the hygrophilous plants of our model) generally show cavitation at higher soil water content than the evergreen [44], which makes them more vulnerable to drought stress; (ii) oaks vary their leaf area index, adjust their stomatal openings, and extend their root system to reach groundwater in such a way as to ensure that evaporation is less than the water supply [10]; (iii) In these areas the water availability has been identified as the major factor shaping vegetation distribution and controlling plant functions [11], [45].

All parameters have been determined, either directly or indirectly, from data available in the literature, referring to annual-mean quantities. Due to the experimental uncertainties, some parameters can only be roughly estimated, while others allow for a precise fit. In Table 1 we summarize the observational constraints that we have used to infer the value of the model parameters.

The potential growth rate, 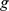, is obtained from the national yield table [46] reporting growth data for a large number of forest species. Yearly-averaged precipitation 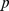 and soil evaporation 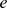 are considered as constants. Their values are readily available from literature [10], [47], [48]. The transpiration ratios 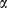 and 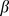 are the mass of water transpired, on average, for the gross production of a unit of biomass. Therefore they are the reciprocal of the water use efficiency (usually expressed as grams of biomass produced per kilogram of water consumed). We assign them a value by taking into account the constraint that non-hygrophilous species have about 25–30% higher water use efficiency than hygrophilous ones [14], [49], [50], [51], and the total transpiration estimates of [52].

The quadratic mortality terms in equations 1 and 2 set the time scale with which the system reacts to perturbations on total biomass. Based on the data in [53] on the recovery time after cuts we estimate 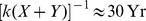, which gives a first guess of the value of 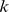. That value is then tuned in such a way that the model reproduces the observed total forest biomass of the study areas (about 6.45 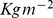 according to [54]).

From the National Forest Inventory Database [54] we derived the metabolic growth and mortality rates of the whole ecosystem, respectively of 2.8–4% and 1.9–2.8% of the total biomass. The items choosen are *Hygrophilous* and *Oak woods* of Lazio. From the FLUXNET database [55] we obtain the average annual soil water content of Castelporziano site for the last 10 years, equal to 37% vol (i.e. about 370 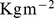), from which the loss factor 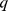 has been calibrated.

Some of the model parameters determine the shape of the functions 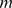 and 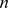, and do not directly correspond to physiological quantities. In most previous works the modeling of mortality is very crude, and it is often reduced to a constant mortality rate. Recent reviews [36], [56] survey the studies on the effect of drought on mortality in different forest systems. We choose parameter values that yield mortality rates roughly consistent with these studies in a realistic range of soil water contents (the shaded gray region in Figure 1).

The half-saturation constants 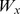 and 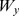 should reasonably be set between the wilting and the saturation points of the soil, that is, between 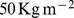 and 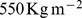 [57]. Their exact value was calibrated in order to have a soil water content at equilibrium close to the observed value and, at the same time, to have the same proportion between hygrophilous and non-hygrophilous species as that observed at the Castelporziano site (roughly 60–70% of Holm oaks [52]).

## Results

### 0.3 Equilibria and their stability

The model embodied by equations (1,2,3) allows for more than one equilibrium, that is, for more than one triplet 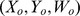 corresponding to zeros of the expressions on the right-hand side. Once an equilibrium has been found, linear stability analysis may be used to determine whether, upon a small external perturbation, the system will return to this equilibrium or not.

For brevity, in the following we use the notation 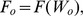 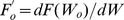, and analogously for the other water-dependent functions. Here we just list the fixed points, and their stability thresholds. The actual stability analysis is summarized in the Appendix S1.

#### Instability of the state with no vegetation

Taking 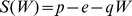, with 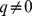, for any choice of the parameters the model always has the trivial equilibrium
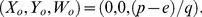
(12)This fixed point is unstable to infinitesimal perturbations if
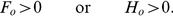
(13)That is, if the net growth rate of at least one of the two species is positive, the state with no vegetation is unstable.

#### Single Species

For most biologically reasonable choices of the functions 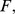 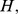 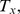 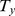 and 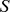, there are two fixed points, one with 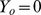, the other with 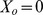. The first one occurs any time that the equation
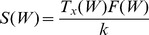
(14)admits a solution for some value 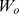 of water content. Then there exists the equilibrium
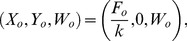
(15)which is meaningful only if 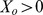, that is, if 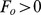. In the Appendix S1 we show that this equilibrium is stable if
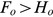
(16)and it is unstable if the inequality is reversed. Therefore, the state with the hygrophilous species alone is stable if, at equilibrium, its growth rate is larger than that of the non-hygrophilous species.

The fixed point with 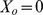 occurs if there is a solution 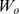 of the equation
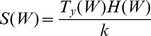
(17)which yields the equilibrium
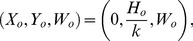
(18)In a way analogous to the previous one, this equilibrium is unstable if
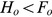
(19)In addition, instability also occurs if
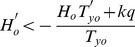
(20)where we have taken 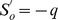, coherently with (8). This inequality may be satisfied because 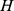 could be a decreasing function of 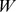 for large values of 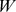. A necessary and sufficient criteria for stability is thus obtained by reversing both inequalities (19) and (20).

#### Coexistence

Equations (1,2,3), may also have a coexistence equilibrium. It is rather straightforward to observe that if 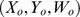 is a coexistence equilibrium, then 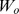 is a solution of
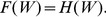
(21)In other words, the intersection of the net growth rates as in Figure 1 is a necessary condition in order to have coexistence. If any such a solution 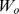 exists, then one finds that the equilibrium biomass densities are
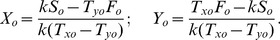
(22)This result is easily extended to the case where the coefficient 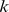 is distinct for the two species. In addition, other reasonable choices for the mortality term would yield coexistence equilibria. We shall not pursue here these generalizations. We observe, however, that with a linear mortality term equations (1,2,3) yield a degenerate coexistence equilibrium which allows for an infinite range of biomasses. This unreasonable result supports our choice of introducing in the model a quadratic death terms corresponding to competition for space.

The expressions (22) are biologically meaningful if both 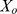 and 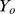 have positive values. This happens when
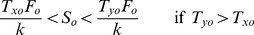
(23)

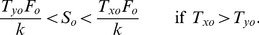
(24)Hence, coexistence is possible if the net equilibrium water input 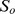 is bracketed between two values which are proportional to the transpiration rates of the 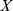 and 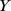 species. As shown in the Appendix S1, when the equilibrium biomass of both species is positive, a necessary condition for stability is
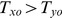
(25)that is, the coexistence is unstable (hence unobservable in practice) unless, at equilibrium, the transpiration rate of the hygrophilous species is larger than that of the non-hygrophilous species. To have a necessary and sufficient stability criterion for coexistence, in addition to (25), it is necessary to check that 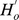 is larger than a complicated expression given in the Appendix S1. However, as this complicated expression is always a negative quantity, if (25) holds and 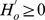, then the coexistence equilibrium is surely stable. This is the case when equations (9,10,11) are used with the parameters of Table 1.

**Table 1:** Observational constraints and values assigned to the model parameters.

*Parameter or*	*Description*	*Assigned*	*Reference*
*Process*		*Value*	
*G*	*Relative growth rate* [*Y r^−^* ^1^]	*0.1*	[*54*]
*W_X_, W_Y_*	*Half saturation constants*	*300; 250*	*Calibrated*
	[Kgm*^−^* ^2^]		
*a; c*	*Mortalities for very low soil*	*0.020;*	*Calibrated according*
	*water content* [Yr*^−^* ^1^]	*0.00*	*to * [*36*], [54], [*56*]
*b; d*	*Mortalities for very high water*	*0.015;*	*Calibrated according*
	*content*[Yr*^−^* ^1^]	*0.075*	*to * [*36*], [54], [*56*]
*W*1, *W*2	*Coefficients that determine the*	*50; 920*	*Calibrated according*
	*shape of mortalities curve*		*to * [*36*], [54], *[56]*
	[Kgm*^−^* ^2^]		
*K*	*Death coefficient in space*	*0.0055*	*Calibrated according*
	*competition terms*		*to* [53]
	[Yr*^−^* ^1^Kg*^−^* ^1^m^2^]		
*P*	*Precipitation* [Kgm*^−^* ^2^]	*780*	[*14]*, [*48*]
*α; β*	*Transpiration ratios*		
	*(dimensionless)*	*900; 600*	[*14*], [49], [50], [51], [*52*]
*E*	*Soil evaporation* [Kgm*^−^* ^2^]	*250*	[*10*]
*Q*	*Loss factor* [Yr*^−^* ^1^]	*0.8*	*Calibrated according*
			*to * [*55*]
*X*+*Y*	*Total Biomass Density*	*6.45*	[*54*]
	[Kgm*^−^* ^2^]		
*f*(*W*)*X*+	*Total Metabolic Growth Rate*	*0.18–0.26;*	[*54*]
*h*(*W*)*Y*	[Kgm*^−^* ^2^ *Y r^−^* ^1^]		
*m*(*W*)*X*+	*Total Water-Induced Mortality*	*0.12–0.18*	[*54*]
*n*(*W*)*Y*	*Rate* [Kgm*^−^* ^2^ *Y r^−^* ^1^]		
*T_X_*(*W*)*X*+	*Total Forest Traspiration*	*207–284*	[*52*]
*T_Y_* (*W*)*Y*	[Kgm*^−^* ^2^ *Y r^−^* ^1^]		
*W*	*Soil water content at the*	370	[*55*]
	*coexistence equilibrium*		
	[Kgm*^−^* ^2^]		
	*Ratio of non-hygrophilous*	70%	[*52*]
	*biomass to total biomass*		
*Y = *(*X*+*Y*)			

Observational constraints and values assigned to the model parameters.

Let us observe that the existence of a stable coexistence equilibrium crucially depends on the net water input 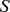, which must lie in the range given by (24). However, at the coexistence equilibrium, neither the soil water content, nor the total biomass density 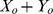 depend on 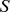, or on the transpiration rates 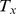 and 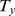. This is a sharp difference with the single species case, where the equilibria change when the water input or the transpiration rates change. Therefore, our model predicts that a forest in a state of coexistence between hygrophilous and non-hygrophilous species is able to maintain a homeostatic equilibrium of total biomass density and soil water content.

**Figure 2:**
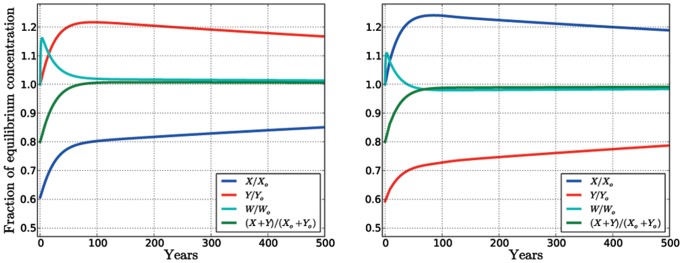
Transient dynamics toward the coexistence equilibrium after a perturbation. The blue, red, cyan and green lines are the ratio of, respectively, the density of the hygrophilous species, the density of the non-hygrophilous species, the soil water content and the total biomass density with their equilibrium concentrations 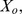 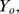 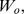
_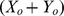. Left panel: the density of the hygrophilous species initially is set at the equilibrium value minus an amount equivalent to 20% of the total equilibrium biomass density. Right panel: the 20% decrement is applied to the density of the non-hygrophilous species, instead. Note that in the left panel the content of water approaches the equilibrium from above; in the right panel the content of water, after the initial increase, drops under the equilibrium value and then approaches the equilibrium from below.

The coexistence may be intuitively justified (see Figure 2) by a sort of facilitation between the two considered species. If we assume that some external cause abruptly decreases the density of the hygrophilous species from its equilibrium values, while leaving the other untouched,the immediate consequence of this perturbation is a rapid increase of the water content in the soil, caused by the decreased total transpiration. This, in turn, causes a rapid increase of the density in both species, because of the more abundant water resource. As the total biomass rises, the water content starts to decline again. So far, the dynamics resembles just a stable equilibrium between water and total biomass. But even when the total biomass has roughly recovered its own equilibrium value, there is still a large disproportion between the two species, with depleted hygrophylous species, and overabundant non-hygrophylous ones. Because of the lower transpiration of the non-hygrophylous, water content is held from returning to its equilibrium value, and remains slightly above it. In this situation the hygrophites have the larger growth rate, and they may increase their biomass (at the expenses of that of the non-hygrophylous) until the full equilibrium is slowly re-established. If, instead, the perturbation had decreased the non-hygrophylous, after the initial sudden water increase, there would be an overall higher water consumption than at equilibrium, and the water content would descend slightly below its equilibrium value. This situation would now favor the depleted hygrophylous, which could slowly regain biomass while the non-hygrophylous would still lose it. Of course, the case of an initial increase of biomass is analogous, and leads again back to equilibrium. While this argument is not a substitute for the rigorous stability analysis discussed in the Appendix S1, we think that it catches the basic ecological mechanism that gives stability to the coexistence equilibrium.

### 0.4 Bifurcation analysis

Although the parameters of Table 1 have been determined on the basis of reliable observational data, they are affected by uncertainty, and by some amount of guesswork. Those determining the shape of the net growth rates 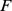 and 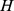 are important, but not crucial: other functional forms could be chosen for 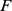 and 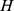 in alternative to those proposed in equations (9,10) and little would change, provided that their graphs still crossed at approximately the same value of 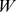, with approximately the same slopes, as shown by the stability analysis in the Appendix S1.

Other parameters are more important in determining the properties of the equilibria of our model. It is therefore important to picture how the equilibria change their stability when these parameters change their value, and discuss the resulting bifurcation diagrams.

#### Bifurcations for changing transpiration ratios

**Figure 3:**
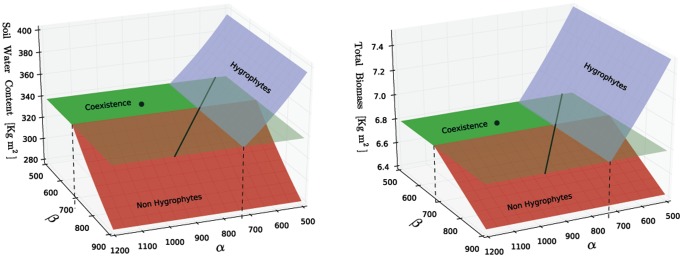
Bifurcation diagram of coexistence and single-species equilibria using 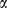 and 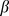 as the control parameters. Left panel: soil water content. Right panel: total biomass. The other parameters are those of Table 1. The red and the blue surfaces represent the stable equilibria with, respectively, only the hygrophilous and the non-hygrophilous species. To avoid visual clutter, unstable branches of these equilibria are not shown. The green plane represents the coexistence equilibrium. It is opaque where the equilibrium is stable and the equilibrium biomasses are positive, and it is transparent where the equilibrium either is unstable or one of the two species has a negative biomass value. The black line lying on the coexistence plane shows where 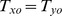. The black dot is the equilibrium state corresponding to the parameters of Table 1. The vertical dashed lines show the position of the bifurcation values 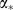, 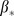.

Plants transpiration data taken in the field are notoriously difficult to measure, in particular if one is interested in year-long averages. Furthermore, in our model the transpiration functions 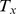 and 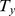 determine many properties of the equilibria. Therefore the values of the non-dimensional parameters 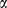 and 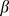, which determine the amount of transpired water on the basis of the metabolic activity of the plants (see eq. 7), are crucial to our analysis. Figure 3 shows a bifurcation diagram in a wide interval of values of 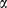 and 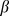, including also regions that probably are physiologically meaningless.

Stable coexistence is possible for relatively high rates 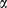 of transpiration of the hygrophilous plants, and relatively low rates 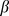 of transpiration of non-hygrophilous plants. This is the opaque green region in the two panels of Figure 3, which appears to be the most physiologically sound. This region is defined by the inequalities 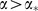 and 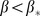 where 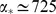, 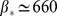 are constants. If either 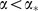 or 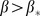 then the coexistence equilibrium loses stability because either 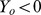 or 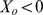 (this is a consequence of the linear stability analysys reported in the Appendix S1, and in particular of equation (1)). In the first case the only stable equilibrium is that where only the hygrophilous species exist. In the second case the only stable equilibrium is that containing only non-hygrophilous species. For either 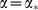 or 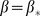 there is a transcritical bifurcation (e.g. [58]) where the coexistence state exchanges its stability with one of the single-species equilibria.

The coexistence equilibrium exists for any value of 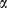 and 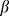, except those that make 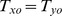, corresponding to singularities in the solutions (22). These special values are identified by the solid black line in the panels of Figure 3. For 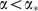 and 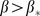 the inequalities (23) hold: the coexistence equilibrium has positive biomass solutions, but it is unstable since 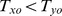. Both the single-species equilibria, however, are stable,which of the two will actually occur depends on the past history of the system. Although the presence of bistability is mathematically interesting, we prefer not to investigate further this regime as we believe it does not represent correctly the physiology of species found in Mediterranean transitional wetland forests. Finally we note that for 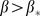, as 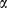 exceeds 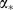, the single-species equilibrium of the hygrophylous species exchanges its stability with a stable, but biologically irrelevant, coexistence equilibrium characterized by 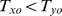 and 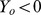; an analogous exchange of stability happens for the non-hygrophylous species for 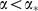 as 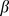 drops below 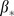.

The state corresponding to the parameters of Table (1), shown by the black dots in Figure 3, is well within the boundaries of stable coexistence. Therefore any conclusion drawn from the model about the coexistence of the two types of species is robust to some level of uncertainty on the numerical values of 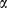 and 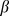.

#### Bifurcations induced by climate change

Let us define as *water supply* the difference 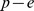 between precipitation and evaporation. Let us also note, from equation (11), that only this difference matters in the model, and not the individual values of precipitation and evaporation. The stability results of section 0.3 show that the water supply crucially enters(through the function 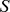 defined in equation 8) in the expression of the single-species and coexistence equilibria (equations 14, 17, 22). We use 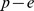 as a control parameter in a bifurcation analysis because the value of 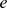 is affect by uncertainty, and, more importantly, because climate models show that the precipitation 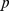 will be significantly altered in the Mediterranean area under climate change scenarios [1].

**Figure 4:**
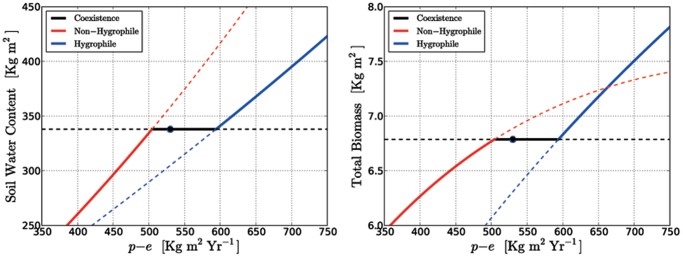
Bifurcation diagram of coexistence and single-species equilibria using 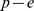 as the control parameter. Left panel: soil water content. Right panel: total biomass. The other parameters are those of Table 1. The 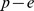 value of the Table is marked by the black dot. Stable equilibria are drawn with solid lines, unstable one with dashed lines.

The bifurcation diagram is shown in Figure 4. Reading the graphs from left to right, i.e. for increasing values of water supply, we note that the non-hygrophilous state is stable and persists until the first bifurcation point, reached at 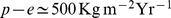. After this threshold the stable state becomes that of coexistence, and the single-species equilibrium looses its stability in a transcritical bifurcation. Increasing further the water supply, for 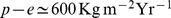 the system undergoes yet another transcritical bifurcation where the coexistence equilibrium becomes unstable, and the single-species equilibrium corresponding to hygrophilous plants becomes stable.

As we mentioned at the end of section 0.3, the soil water content and the total biomass density at the coexistence equilibrium are independent of the water supply. What changes for varying water supply is the proportion of the hygrophilous and non-hygrophilous species. Rearranging equations (22), we may express the fraction of hygrophylous and non hygrophylous species biomass with respect to the total biomass as
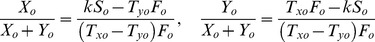
(26)where 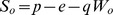. When 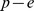 is as low as to make 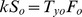 (the leftmost bifurcation point in Figure 4) then 100% of the biomass is made of non-hygrophites. For rising water supply the fraction of hygrophites rises at the expenses of the non-hygrophites, until, for 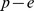 sufficiently high as to make 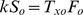, 100% of the biomass is composed by hygrophites (this is the rightmost bifurcation point in Figure 4).

#### Robustness of the regime-shift scenarios induced by climate change

The above analysis implies the possibility of a regime shift accompanied by a dramatic loss of biodiversity if climate change drives 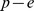 beyond the the bifurcations shown in Figure 4, thus destabilizing the coexistence equilibrium. Therefore it is important to assess if such a scenari would be robust despite the uncertainties in the values of the parameters. As we have argued above, the most important parameters in this respect are the transpiration coefficients 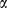 and 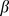.

As shown by equations (26), the destabilization of a coexistence equilibrium for varying 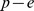 happens by reducing down to zero the biomass density of one of the two types of trees. The inequalities (24) mark the boundaries of this stability region, and involve 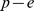 (contained in 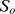), and 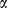 and 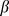 (contained in 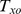 and 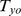, respectively).

**Figure 5:**
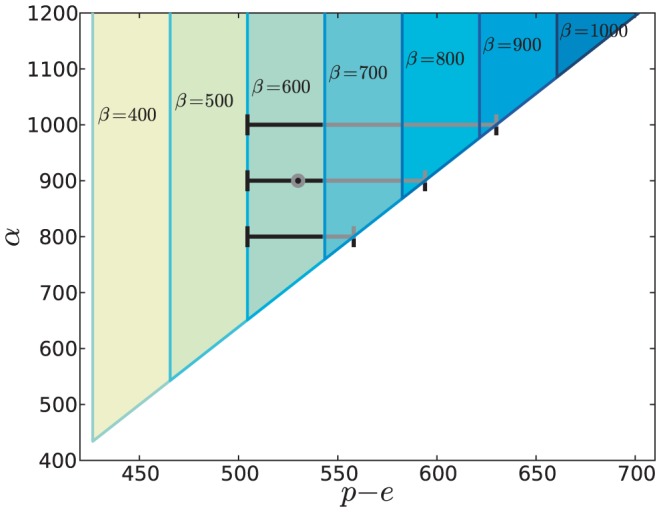
Ranges of stable coexistence depending on 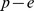 and 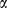 at seven distinct values of 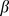. The black dot shows the equilibrium corresponding to the parameters of Table 1. The horizontal segment containing the dot is the same coexistence interval appearing in Figure 4. The other two horizontal segments show the stable coexistence 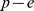 intervals for 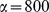 and 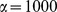 within the 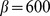 wedge. For every fixed value of 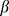, the range of stable coexistence in the 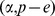 plane forms a triangular wedge.

Figure 5 is a graphical depiction of the boundaries given by (24). For any fixed value of 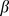, the stability region of coexistence is represented by a wedge in the 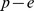 vs 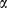 plane. The position of the left side of this wedge, corresponding to the bifurcation where coexistence is replaced by the non-hygrophylous species alone, is independent of 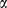. The right side, corresponding to coexistence replaced by just the hygrophylous, is a straight line (the same for all values of 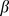) that slopes in such a way that the coexistence interval becomes larger as 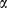 increases. Figure 5 shows with different colors the stability wedges for seven distinct values of 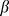, piling-up on top of each other.

It is evident that the possibility of a regime-shift as 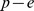 changes is always present. Uncertainties in the value of the transpiration rates are reflected as uncertainties in the exact position of the bifurcation threshold. Nevertheless, the boundaries of the 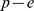 stability intervals in Figure 5 are always attained at values reasonable for a Mediterranean forest.

## Discussion and Conclusions

We believe that the knowledge of the basic physiological processes underlying the adaptive strategies of plants has just reached a point where it is possible to develop quantitative, dynamic models which are simple enough to be amenable to analysis, and yet rich enough in ecological complexity to resemble reality. We propose such model as a research tool for the ecological analysis of coexistence of distinct tree types in forested wetlands. The model, through a mathematical representation of a set of physiological mechanisms, should represent the essential ecological features of ecotones where water can be both a limiting resource and a chronic stressor.

One of the key features of the model is the explicit modeling of mortality as a water-dependent process, which may either favor the hygrophilous species and hamper the non-hygrophilous ones, or viceversa, depending on the soil water content. The other ingredients that allow for a stable coexistence are the interplay of higher water use efficiency of the non-hygrophilous species, and higher transpiration rates of the hygrophilous ones.

Although the mechanism of coexistence appears to be robust, and we find it in large regions of the parameter space, the bifurcation analysis shows that coexistence is possible only within a somewhat narrow interval of water supply values (here defined as the differencebetween precipitation and evaporation: 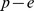). Using the model with the best fit of the parameters (Table 1), we find that the extinction of the hygrophilous species happens at 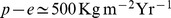. According to the expected climate scenarios for the Mediterranean basin [1], [2], such a minimum threshold of water supply could be reached in the second half of the current century. Thus our model forecasts a potential dramatic shift towards non-hygrophilous forest communities that would result in a loss of biodiversity in the coastal plain woodlands, exacerbated by the strong local endemism found in those sites. Of course, uncertainties in the value of the transpiration ratios, 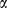 and 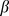, are reflected as uncertainties in the exact position of the bifurcation threshold. Nevertheless, for a wide range of the transpiration ratios the bifurcation threshold lies at values of 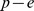 which are in the range of the projected climate changes (Figure 5).

It is important to highlight that the nature of the bifurcations that destabilize the state of coexistence (transcritical rather than fold bifurcations) is such that hysteresis phenomena are ruled out. Therefore, if the value of 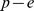 moved outside the range of stable coexistence and then returned within that range after the local extinction of one of the two group of species, then, according to the model, the ecosystem could be recolonized if the extinct species were reintroduced.

**Figure 6:**
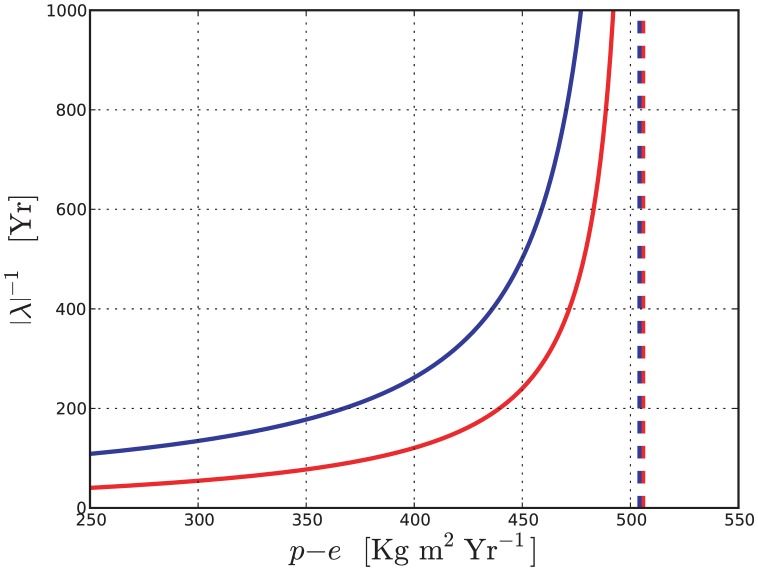
Reciprocal of the eigenvalue closer to zero of the linearization around the stable equilibrium containing only non-hygrophites as a function of 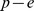 (solid lines). The blue line is computed using the parameters of Table 1. The red line is computed with the same parameters except for 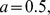 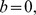 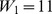. The vertical blue and red dashed lines show the position of the bifurcation. For larger values of 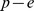 the single species equilibrium is unstable and the coexistence equilibrium is stable.

Even more important, in view of the precipitation decrease projected by regional climate models in the Mediterranean area, would be to quantify the characteristic time required for the extinction of the hygrophilous species. Unfortunately, the available data (discussed in section (0.2)) is insufficient to constrain the shape of the functions that appear in equations (1–3) well enough to give a reliable quantitative description of the transient dynamic when the system is brought out of equilibrium. An example of this statement is given in Figure 6.

A rough estimate of the extinction time of the hygrophylous species when the precipitation is abruptly decreased below the lower threshold of stable coexistence is the reciprocal of the eigenvalue closer to zero of the linearization around the equilibrium containing only non-hygrophylous species (see the Appendix S1 for the mathematical details). We compute this quantity for two distinct sets of parameters, differing just for the shape of the hygrophylous species mortality function 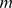. Both sets give approximately the same values of biomass density, soil water content and bifurcation points at the stable coexistence equilibrium corresponding to the current precipitation level (with a relative discrepancy of less than 7%). Therefore they both yield an acceptable model of the present state of the woods discussed in section 0.2. However, the strongly different mortality of the hygrophytes at low soil water content makes the transient dynamics much more rapid for one set of parameters than for the other.

The model, of course, has some assumptions and simplifications that may limit the reliability of its predictions. The state of coexistence is just the result of competitive interactions. No facilitation is explicitly included in the model, although the stability mechanism described above, where the overabundance of one type of species sets a water level that favors the growth of the other, may be seen as some sort of facilitation mechanism (for a review on positive plant interactions see [33]). Among the many ecological interactions neglected by the model there certainly may be some that increase the resilience of the coexistence states to variations of the hydrological regime. [59]. Having set all the parameters as time-independent constants is another gross simplification. Therefore, the model is unable to take into account any feature or adaptation of the ecosystem linked to the seasonal cycle. We plan to address this shortcoming in our future work.

In the physiologically plausible range of parameters the model lacks multiple stable equilibria. This implies that even extremely large perturbations can not produce regime shifts, if the external parameters remain the same. Whether this is a genuine property of these kind of ecosystems or an oversimplification of the model remains to be seen.

Finally, the distinction between hygrophilous and non hygrophilous species is quite coarse and may not be perfectly representative of the Mediterranean woodlands. However, the model does not immediately generalize to three or more groups characterized by distinct sensitivities to the water stress. At the moment it is not clear to us whether such a generalization should necessarily introduce an explicit description of space, for example along the lines suggested by Tilman [30], or if a satisfactory spatially-implicit formulation might be found.

In spite of all these cautionary notes, our model is able to reproduce the observed data, and it shows that rainfall availability has a critical effect on the ability of Mediterranean wetlands to maintain species coexistence and hence to sustain biodiversity. This finding calls for an urgent adaptation and mitigation response aimed at protecting and enhancing the hydrological balance through specific interventions, both in terms of water management and regulatory mechanisms, to prevent human pressure on water resources in proximity of wetland forested areas. Our work confirms the importance of using dynamic, deterministic models for identifying vulnerabilities and thresholds while assessing the impacts of climate change. We believe that in the future approaches like this model will have a wider use because they provide simple and clear instruments to policy makers and planning institutions for decision making.

## Supporting Information

Appendix S1 **Equilibria stability analysis.**
(PDF)
