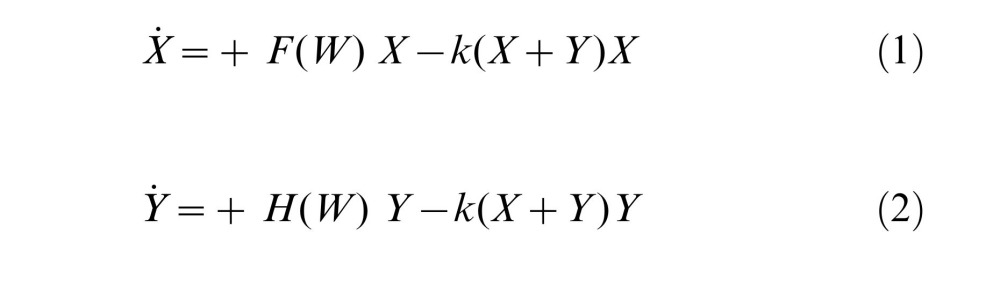# Correction: Climate Change Threatens Coexistence within Communities of Mediterranean Forested Wetlands

**DOI:** 10.1371/annotation/eb12a04d-53fc-4479-a808-fbb6f4123712

**Published:** 2013-10-29

**Authors:** Arianna Di Paola, Riccardo Valentini, Francesco Paparella

An error was introduced in the preparation of this article for publication. Equations 1 and 2 in the Materials and Methods section under the subheading "0.1 Model formation" should not begin with a minus sign. Please view the corrected equations 1 and 2 here: