# Supplementary material for: Climate Change Threatens Coexistence within Communities of Mediterranean Forested Wetlands
Source: PLoS One. 2012 Oct 8;7(10):e44727. doi: 10.1371/journal.pone.0044727 (PMC3466281; doi:10.1371/journal.pone.0044727)
Supplement: Appendix S1 — Equilibria stability analysis. (PDF) [file pone.0044727.s001.pdf]

Appendix

For autonomous systems of ordinary differential equations, such as (1-3) the linear stability properties of an equilibrium are determined by the eigenvalues of the Jacobian matrix evaluated at the equilibrium point [58]. In particular, the equilibrium is linearly stable when all the eigenvalues have negative real part, and it is unstable when at least one of them has a positive real part. We denote with (X_o, Y_o, W_o) the equilibrium. We also define $F_o = F(W_o)$, $F'_o = dF(W_o)/dW$, and analogously for the other water-dependent functions appearing in equations (1-3).

No vegetation. The Jacobian matrix evaluated at the equilibrium (12) is

$$J_o = \begin{pmatrix} F_o & 0 & 0 \\ 0 & H_o & 0 \\ -T_{xo} & -T_{yo} & S'_o \end{pmatrix}.$$

In this case the eigenvalues coincide with the entries on the main diagonal. Generally we expect $S'_o < 0$ which means that the loss of soil water (e.g. through percolation) increases when the soil water content increases. Therefore the instability condition (13) follows.

Hygrophilous Species Equilibrium. The Jacobian matrix evaluated at the equilibrium (15) is

$$J_o = \begin{pmatrix} -F_o & -F_o & k^{-1} F_o F'_o \\ 0 & H_o - F_o & 0 \\ -T_{xo} & -T_{yo} & S'_o - k^{-1} F_o T'_{xo} \end{pmatrix}$$

In this case the characteristic polynomial of J_o is easily factored as the product of a first degree and a second degree polynomial:

$$P(\lambda) = (H_o - F_o - \lambda) \left[k^{-1} F_o \left(-k S'_o + T_{xo} F'_o + T'_{xo} F_o \right) + \left(-S'_o + k^{-1} F_o T'_{xo} + F_o \right) \lambda + \lambda^2 \right]$$

The second-degree polynomial has strictly positive coefficients. In fact $F_o > 0$ is required to have a positive biomass concentration at equilibrium, $T'_{xo}, F'_o > 0$ and $S'_o < 0$ as a result of the assumptions of monotonicity discussed in section (2.1). Therefore the roots of the second degree polynomial have strictly negative real part. The root of the first degree polynomial yields the eigenvalue

$$\lambda = H_o - F_o.$$

whose sign determines the stability of the equilibrium.

Non-Hygrophilous Species Equilibrium. The analysis is completely analogous to the hygrophilous case, and the characteristic polynomial is

$$P(\lambda) = (F_o - H_o - \lambda)\left[k^{-1}H_o\left(-kS'_o + T_{yo}H'_o + T'_{yo}H_o\right) + \left(-S'_o + k^{-1}H_oT'_{yo} + H_o\right)\lambda + \lambda^2\right].$$

However, in this case the sign of H'_o is not known beforehand. If $H'_o < T_{yo}^{-1}\left(kS'_o - T'_{yo}H_o\right)$ then the second-degree polynomial has a negative constant term and positive first- and second-degree coefficient. Therefore it has a positive and a negative real root, which implies instability.

Coexistence Equilibrium. The Jacobian matrix at the coexistence equilibrium (21), (22) is

$$J_o = -\begin{pmatrix} kX_o & kX_o & -X_oF'_o \\ kY_o & kY_o & -Y_oH'_o \\ T_{xo} & T_{yo} & Y_oT'_{yo} + X_oT'_{xo} - S'_o \end{pmatrix}.$$

The characteristic polynomial of J_o is not easily factored. Although the Cardano-Lagrange formulae would allow to write down explicitly the eigenvalues of J_o, the resulting expressions are rather unwieldy, and it is not straightforward to determine the sign of the real part of the eigenvalues. Therefore we use an indirect approach to determine the stability of a coexistence equilibrium. First we observe that the determinant of J_o is

$$\Delta = -k(T_{xo} - T_{yo})(F'_o - H'_o)X_oY_o. \tag{1}$$

Recalling that the determinant is the product of the three eigenvalues of J_o, it is clear that a necessary condition for stability is $\Delta < 0$. All the quantities appearing in the expression above are positive, except for H'_o which may have any sign. However, $(F'_o - H'_o) > 0$, because, according to the assumptions discussed in section (2.1), we have $F(W) < H(W)$ for $W < W_o$ and $F(W) > H(W)$ for $W > W_o$. Therefore, the necessary condition (25) follows. In order to rule out the case of two eigenvalues both with positive real part, it is necessary to supplement the criterion (25) with an additional inequality. This is accomplished by the use of the Routh-Hurwitz criterion. A review of the criterion is well beyond the scope of this paper. See exemples [1] for an exhaustive treatment of the subject, or [2] for a simple exposition with examples taken from biological problems. In our case, after straightforward but long and tedious calculations, we obtain that the equilibrium is stable if and only if $\Delta < 0$ and

$$
\begin{aligned}
H_o' \quad > \quad & -\left(T_{yo}Y_o^2T_{yo}' + T_{yo}X_oY_oT_{xo}' + kT_{yo}Y_o^2 + kT_{xo}X_oY_o + qT_{yo}Y_o\right)^{-1} \\
& \left[F_o'\left(T_{xo}X_oY_oT_{yo}' + T_{xo}X_o^2T_{xo}' + kT_{yo}X_oY_o + kT_{xo}X_o^2 + qT_{xo}X_o\right) + \right. \\
& +kY_o^3T_{yo}'^2 + kX_oY_o^2T_{yo}'^2 + 2kX_oY_o^2T_{xo}'T_{yo}' + 2kX_o^2Y_oT_{xo}'T_{yo}' + \\
& +k^2Y_o^3T_{yo}' + 2k^2X_oY_o^2T_{yo}' + 2kqY_o^2T_{yo}' + k^2X_o^2Y_oT_{yo}' + \\
& +2kqX_oY_oT_{yo}' + kX_o^2Y_oT_{xo}'^2 + kX_o^3T_{xo}'^2 + k^2X_oY_o^2T_{xo}' + \\
& +2k^2X_o^2Y_oT_{xo}' + 2kqX_oY_oT_{xo}' + k^2X_o^3T_{xo}' + 2kqX_o^2T_{xo}' + \\
& \left. +k^2qY_o^2 + 2k^2qX_oY_o + kq^2Y_o + k^2qX_o^2 + kq^2X_o\right]
\end{aligned}
$$

where we have set $-S_o' = q > 0$. Although this expression is too long to be of any direct practical use, it still carries useful information if one observes that, at least for the biologically relevant case of $X_o, Y_o > 0$, the right-hand side is a negative quantity. Therefore, for positive values of the equilibrium biomass densities, if one finds $\Delta < 0$ and a positive value of H_o', then the coexistence fixed point is stable.

References

[1] Gantmacher, FR (2000). The Theory of Matrices vol. 2. American Mathematical Society, Providence, Rhode Island, USA.

[2] Otto SP, Day T (2007). A biologist's guide to mathematical modeling in ecology and evolution. Princeton University Press, Princeton, New Jersey, USA.
